# Defining disease severity in atopic dermatitis and psoriasis for the application to biomarker research: an interdisciplinary perspective

**DOI:** 10.1093/bjd/ljae080

**Published:** 2024-02-29

**Authors:** Ravi Ramessur, Nick Dand, Sinéad M Langan, Jake Saklatvala, Marie-Christine Fritzsche, Suzi Holland, Bernd W M Arents, Helen McAteer, Andrew Proctor, David McMahon, Michelle Greenwood, Alena M Buyx, Tamara Messer, Nina Weiler, Alexandra Hicks, Peter Hecht, Stephan Weidinger, Matladi N Ndlovu, Dai Chengliang, Matthias Hübenthal, Alexander Egeberg, Lavinia Paternoster, Lone Skov, Elke M G J De Jong, Maritza A Middelkamp-Hup, Satveer K Mahil, Jonathan N Barker, Carsten Flohr, Sara J Brown, Catherine H Smith

**Affiliations:** St John’s Institute of Dermatology; Department of Medical and Molecular Genetics, School of Basic and Medical Biosciences, Faculty of Life Sciences & Medicine, King’s College London, London, UK; Department of Medical and Molecular Genetics, School of Basic and Medical Biosciences, Faculty of Life Sciences & Medicine, King’s College London, London, UK; London School of Hygiene & Tropical Medicine, London, UK; Department of Medical and Molecular Genetics, School of Basic and Medical Biosciences, Faculty of Life Sciences & Medicine, King’s College London, London, UK; Institute of History and Ethics in Medicine, TUM School of Medicine; Department of Science, Technology and Society, School of Social Sciences and Technology, Technical University of Munich, Munich, Germany; Eczema Outreach Support, Linlithgow, UK; Dutch Association for People with Atopic Dermatitis, Nijkerk, the Netherlands; The Psoriasis Association, Northampton, UK; National Eczema Society, London, UK; Irish Skin Foundation, Dublin, Ireland; Irish Skin Foundation, Dublin, Ireland; Institute of History and Ethics in Medicine, TUM School of Medicine; Department of Science, Technology and Society, School of Social Sciences and Technology, Technical University of Munich, Munich, Germany; EURICE – European Research and Project Office GmbH, St. Ingbert, Germany; EURICE – European Research and Project Office GmbH, St. Ingbert, Germany; Immunology & Inflammation Research Therapeutic Area, Sanofi, Cambridge, MA, USA; Public Private Partnerships, Sanofi Partnering, Frankfurt, Germany; Department of Dermatology, Venerology and Allergology, University Hospital Schleswig-Holstein, Kiel, Germany; UCB Pharma, Brussels, Belgium; Translational Medicine Department, UCB S.A., London, UK; Department of Dermatology, Quincke Research Center, University Hospital Schleswig-Holstein, Campus Kiel, Kiel, Germany; Department of Dermatology, Bispebjerg Hospital, Copenhagen, Denmark; Department of Clinical Medicine, University of Copenhagen, Copenhagen, Denmark; MRC Integrative Epidemiology Unit at the University of Bristol, Bristol, UK; Population Health Sciences, Bristol Medical School, Bristol, Bristol, UK; Department of Clinical Medicine, University of Copenhagen, Copenhagen, Denmark; Department of Dermatology and Allergy, Copenhagen University Hospital – Herlev and Gentofte, Copenhagen, Denmark; Department of Dermatology, Radboud University Medical Centre, Nijmegen, the Netherlands; Department of Dermatology, Amsterdam Public Health, Infection and Immunity, Amsterdam UMC, Academic Medical Center, University of Amsterdam, Amsterdam, the Netherlands; St John’s Institute of Dermatology; Department of Medical and Molecular Genetics, School of Basic and Medical Biosciences, Faculty of Life Sciences & Medicine, King’s College London, London, UK; St John’s Institute of Dermatology; Department of Medical and Molecular Genetics, School of Basic and Medical Biosciences, Faculty of Life Sciences & Medicine, King’s College London, London, UK; Unit for Paediatric and Population-Based Dermatology Research, St John’s Institute of Dermatology, Guy’s and St Thomas’ NHS Foundation Trust and King’s College London, London, UK; Centre for Genomic and Experimental Medicine, University of Edinburgh, Edinburgh, UK; Department of Dermatology, NHS Lothian, Edinburgh, UK; St John’s Institute of Dermatology

## Abstract

More severe atopic dermatitis and psoriasis are associated with a higher cumulative impact on quality of life, multimorbidity and healthcare costs. Proactive, early intervention in those most at risk of severe disease may reduce this cumulative burden and modify the disease trajectory to limit progression. The lack of reliable biomarkers for this at-risk group represents a barrier to such a paradigm shift in practice. To expedite discovery and validation, the BIOMarkers in Atopic Dermatitis and Psoriasis (BIOMAP) consortium (a large-scale European, interdisciplinary research initiative) has curated clinical and molecular data across diverse study designs and sources including cross-sectional and cohort studies (small-scale studies through to large multicentre registries), clinical trials, electronic health records and large-scale population-based biobanks. We map all dataset disease severity instruments and measures to three key domains (symptoms, inflammatory activity and disease course), and describe important codependencies and relationships across variables and domains. We prioritize definitions for more severe disease with reference to international consensus, reference standards and/or expert opinion. Key factors to consider when analysing datasets across these diverse study types include explicit early consideration of biomarker purpose and clinical context, candidate biomarkers associated with disease severity at a particular point in time and over time and how they are related, taking the stage of biomarker development into account when selecting disease severity measures for analyses, and validating biomarker associations with disease severity outcomes using both physician- and patient-reported measures and across domains. The outputs from this exercise will ensure coherence and focus across the BIOMAP consortium so that mechanistic insights and biomarkers are clinically relevant, patient-centric and more generalizable to current and future research efforts.

## Teaser

The discovery of severity biomarkers is crucial for facilitating a proactive, risk-stratified approach in the management of atopic dermatitis and psoriasis. This review describes the multifaceted aspects and challenges in defining disease severity in the context of biomarker research. Important considerations when analysing datasets across diverse study types are summarized to help expedite biomarker development for inflammatory skin diseases in the future.

Linked Article: Bentz and Weisshaar *Br J Dermatol* 2024; **191**:3–4.

## Introduction

Atopic dermatitis (AD) and psoriasis are common, chronic inflammatory disorders with a substantial disease burden.^1,2^ This burden depends on multiple factors including the body sites affected, intensity and duration of skin inflammation, the presence of associated comorbidities and the consequent impact on the person affected and those around them. In general, the more severe the skin disease, the greater the burden.^3^ At present, treatment paradigms tend to be reactive to the prevailing disease state, with minimal emphasis on disease prevention, no routinely used strategies to identify individuals most at risk of greater disease burden, a trial-and-error approach to treatment choice and consequently potentially avoidable impact on quality of life.

Biomarkers that associate with disease severity at a point in time may serve as useful diagnostic biomarkers of disease severity (e.g. for entry into clinical trials) or monitoring biomarkers (to objectively track disease activity over time). Furthermore, the development of prognostic biomarkers (biomarkers measured early in the disease course that provide information about the likelihood of progression to more severe and/or difficult to control disease) is crucial to facilitate a more proactive, risk-stratified approach, both in clinical trials and routine practice. This, in turn, could expedite drug development and enable early intervention to potentially modify the disease course (e.g. by shortening time to longer-term remission or prevention of comorbidities).^4,5^ The BIOMarkers in Atopic Dermatitis and Psoriasis (BIOMAP) consortium (www.biomap-eu.imi), a large-scale pan-European, public–private, interdisciplinary research initiative, has curated and harmonized clinical and molecular data from patient collections, disease registers, epidemiological studies and clinical trials with reference to a glossary of clinical phenotypes and outcomes.^6^ This rich resource, comprising multiple data types, is expected to expedite biomarker discovery and validation.

To ensure future clinical utility and adoption, the purpose of any biomarker needs to be explicitly considered by all relevant stakeholders. Therefore, it is important to define what is meant by severe disease. Despite the existence of widely used diagnostic criteria^7,8^ and multiple severity measures,^9,10^ there is no unified validated definition of severe disease in either AD or psoriasis.^11^ Here, we leverage broad interdisciplinary expertise across the BIOMAP consortium to describe the multifaceted aspects of disease severity, along with related instruments and measures that might be used for assessment. We reference and prioritize commonly used definitions of severe disease and important variables to consider when analysing datasets across diverse study types, with the overall aim of expediting biomarker development that is relevant to future drug development and population health in the field of inflammatory skin disease.

## Methods

Using a framework for discussion informed by previous systematic reviews of biomarkers in AD and psoriasis,^12,13^ we convened a series of meetings with a multidisciplinary research group drawing on BIOMAP participants from academia and industry including 13 clinical experts, 9 scientists, 2 bioinformaticians and 6 representatives from patient organizations. We described domains of disease severity and approaches to assessment,^14^ and then surveyed the datasets within BIOMAP and mapped all disease severity instruments to these domains. Findings were cross-referenced with existing international consensus [e.g. Harmonising Outcomes for Eczema (HOME),^15,16^ International Psoriasis Council (IPC),^17^ International Dermatology Outcome Measures Initiative (IDEOM),^18^ regulatory authorities (US Food and Drug Administration, the European Medicines Agency, the National Institute for Health and Care Excellence)],^14,19^ a targeted literature search and BIOMAP multidisciplinary group expert opinion. The literature search was conducted in MEDLINE, searching for articles relating to defining disease severity in AD and psoriasis from the inception of the database to 30 July 2023 [search terms (‘atopic dermatitis’ OR ‘psoriasis’) AND (‘severity’ OR ‘severe’ OR ‘measures’ OR ‘tool’ OR ‘instrument' OR ‘Assessment’ OR ‘Classification’ OR ‘Index’ OR ‘Score’ OR ‘Rating’ OR ‘Scale’ OR ‘Criteria’ OR ‘Grading’ OR ‘Quality of Life’ OR ‘Disease Burden’ OR ‘Health Outcomes’ OR ‘Patient-Reported Outcomes’)]. Only published articles in the English language of any article type were reviewed. We annotated the complete set of severity instruments according to the following (often overlapping) criteria: (i) subject to formal international consensus and (ii) expert opinion and/or regulatory reference standard. These criteria were used to indicate priority definitions of severe disease and related measures, which the group agreed would best capture the aspect of disease severity in each respective domain and recommend for use in analyses when available. Interrelationships across domains were highlighted in addition to key factors to consider when analysing datasets within the biomarker development pipeline.

## Results

### Describing the severity of atopic dermatitis and psoriasis: domains for consideration

**Figure 1:**
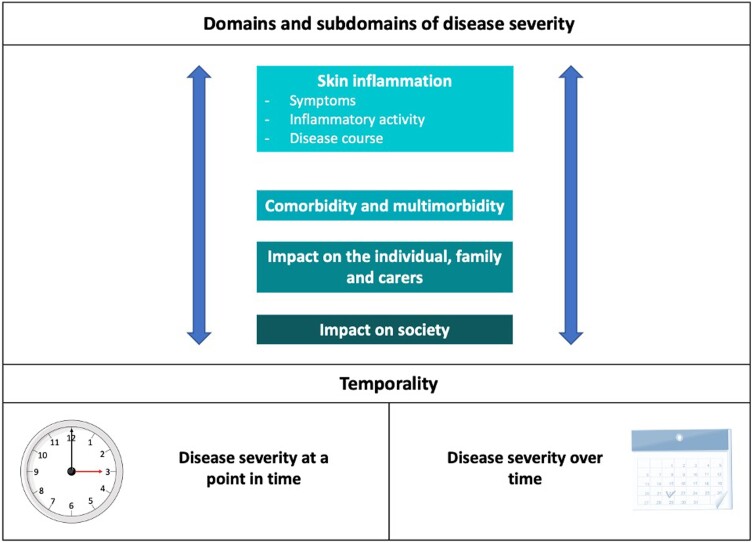
Conceptual overview of domains and subdomains of disease severity in atopic dermatitis and psoriasis.

To agree on a common framework for investigation, key domains of disease severity were defined by our multidisciplinary research group (Figure 1) and with reference to relevant existing published consensus.^17,20^

### Skin inflammation

Skin inflammation is the hallmark of both AD and psoriasis. The key subdomains comprise symptoms, inflammatory activity (character, extent and sites of involvement at a point in time) and disease course (duration, pattern of activity over time, and response or resistance to treatment). These subdomains are not discrete; rather they are overlapping and interdependent. Nevertheless, they are useful to consider, and relevant to biomarker development. Firstly, severity in any one subdomain may be different in another, for example hand eczema is typically associated with severe symptoms even though it is limited in extent. Secondly, the underpinning mechanisms (and subsequent biomarker purpose) in each subdomain may have significant differences.

### Symptoms

Symptoms of skin inflammation, such as itch, bleeding and pain, may be captured through patient-reported outcome measures either in isolation (e.g. Itch Numeric Rating Scale), as part of a composite tool (e.g. SCORing Atopic Dermatitis) or, in the case of itch, through semiobjective measures such as movement monitors and wearable devices.

### The inflammatory activity

The most commonly used instruments for assessing inflammatory activity in both trials and clinical practice still rely on a visual assessment of the skin – either by a healthcare professional (often referred to as physician- or clinician-­reported measures) or by the person affected. A wide range of instruments have evolved and are available, but relatively few are used in the context of trial and/or routine clinical practice [e.g. Psoriasis Area and Severity Index (PASI),^21^ Eczema Area and Severity Index (EASI)].^22^ Inter- and intraindividual variation, in addition to inherent problems with many of the instruments themselves, limits comparison across different studies and healthcare systems. Different measures of inflammatory activity are not easily comparable, despite previous interpretability efforts in AD^23,24^ and psoriasis.^25^ Emergent image-based artificial intelligence tools show promise as objective tools and may overcome some of the limitations of conventional methods in the future.^26^

### Disease course

Inflammation varies acutely during disease flares, periodically during the life course of affected individuals and/or in relation to external triggers.^1,27^ Certain clinical subtypes of AD and psoriasis tend to have a characteristic natural history, for example childhood AD may resolve by adolescence/adulthood^28^ and guttate psoriasis may resolve or progress to chronic plaque disease.^29^ Seasonal variation related to the effects of temperature, humidity, ultraviolet radiation, aeroallergens, superimposed infection [both type (e.g. eczema herpeticum in AD) and frequency], environmental and societal factors all potentially influence the disease course in any individual. Many of these factors are difficult to quantify (and/or not recorded) and are therefore challenging to account for in subsequent analyses. Therapeutic intervention will also influence, and perhaps even permanently modify, the ‘natural’ disease course.

The measures used to capture ‘disease course’ will depend on the purpose and timeframe of interest. Measures such as Recap of atopic eczema (RECAP)^30^ and Atopic Dermatitis Control Tool (ADCT)^31^ have been developed specifically to quantify disease control – an aspect of disease course – in the short term (1 week). Healthcare use can be a useful proxy for disease course over more prolonged periods of time, with advanced treatments and/or more intensive healthcare settings indicating sustained or more severe disease. Indeed, the IPC has recommended use of a dichotomized definition of psoriasis severity where the need for systemic therapy is the defining criterion for severe disease.^17^ However, healthcare resource use is influenced by many factors beyond skin inflammation itself, including the individual (patient’s perception of their own disease, knowledge and access to healthcare, cultural and economic considerations) and societal factors (healthcare provision, etc.). Use of and access to health resources consequently varies by geographical location, which limits the direct comparability on an international scale. Eligibility thresholds for advanced systemic treatments (e.g. biologics or small-molecule inhibitors) are also subject to change over time; therefore, this variability needs to be accounted for when using systemic treatment use as a measure of disease severity. For example, biologic use in psoriasis has rapidly increased over the last decade, with increasing safety data and confidence in the prescribing community in addition to improved access to these treatments.

### Comorbidity and multimorbidity

The mechanisms underpinning the associations between atopic or psoriatic skin inflammation and their respective morbidities are incompletely understood, and may be morbidity-specific.^1^ Certain conditions, e.g. AD and asthma or psoriasis and psoriatic arthritis, share genetic risk factors^32,33^ and therefore appear likely to be driven by shared disease biology. For example, the established effectiveness of agents targeting the interleukin (IL)-4/IL-13 pathway in both diseases supports the concept of shared biology between these traits.^34^ Other comorbidities may influence skin inflammation directly, for example obesity and cardiovascular disease have been reported to be causally related to psoriasis risk.^35,36^ Prevalent mental health disorders such as depression and anxiety are also associated with more severe inflammatory skin disease, but these disorders may be the result of severe inflammatory skin disease rather than the cause.^37,38^

When present, comorbidities contribute to the overall impact of AD or psoriasis on affected individuals. However, when investigating biomarkers of skin disease severity, it is important to consider the mechanistic differences in their link with AD and psoriasis if comorbidities are used as a proxy measure of more severe skin inflammation.

### Impact on the individual, family, friends and carers

For the individual affected, both AD and psoriasis can impact on physical, psychological and/or social wellbeing and related economic health.^39^ The exact nature and degree of this impact will depend not only on the severity of the skin inflammation, but also on a host of other factors including age, stage in life and available support, socioeconomic status and access to care. For example, AD and psoriasis in childhood can adversely affect schooling and ability to learn; involvement at high-impact sites, such as the hands and feet, influences functional status and ability to work, and chronic sleep deprivation is likely to contribute to prevalent depression in AD.^40^ Healthcare interactions (e.g. multiple attendances for phototherapy, frequent clinic visits in the context of treatment refractory disease), burden of treatments (e.g. time spent applying topical treatments) and treatment-related side-effects all contribute to the burden. Measures such as the Treatment Satisfaction Questionnaire for Medication have been specifically developed to capture the impact of treatments in chronic disease.^41^

There is also increasing recognition and assessment of the impact on family, friends and carers.^42,43^ Multiple instruments are used to capture this impact – both on the individual and those around them, e.g. Psoriasis Family Index, Family Dermatology Life Quality Index, Dermatitis Family Impact Questionnaire and Family Reported Outcome Measure (FROM-16).^44–47^ The impact of AD and psoriasis compounds over the life course of those affected, leading to a substantial cumulative life burden.^48,49^ It is important to consider the complexity of factors influencing these measures in the context of biomarker development.

### Impact on society

The wider societal impact of AD and psoriasis includes loss of the ability to fully function as part of society (for both the individual and those around them), loss of productivity owing to impact on employment (economic impact) and consumption of healthcare resources, especially when the symptoms are poorly controlled.^50,51^ In the context of biomarkers, measures of this societal impact are more relevant at the implementation and adoption stage. In addition, discussions about the ethical and social implications of using disease severity biomarkers and how such implications should guide medical decisions and actions in clinical and public health settings are particularly important in the implementation phase.

### Additional considerations

The described domains overlap and do not encompass all aspects of disease severity. Infections for example, particularly those associated with AD, act both as triggers for disease flare-ups and as consequences of inadequately controlled skin inflammation. Thus frequency and type of infections, such as eczema herpeticum and *Staphylococcus aureus* infection serve as additional potential proxies for disease severity. We do not explicitly include treatment-­related toxicity such as osteoporosis with corticosteroids or renal dysfunction with ciclosporin. These drug-related toxicities are important, strongly associate with more severe disease and contribute to the overall burden. They are multifactorial in origin and – as exemplified by observed liver fibrosis in the methotrexate-exposed population – may not be primarily driven by the drug. With improved mechanistic understanding, the distinction between association and causation will become clearer and so we would expect this overall schema of disease severity domains and measures to evolve over time.

## Mapping measures of disease severity to key domains

### Sources of severity measurements

BIOMAP datasets represent data available across Europe for AD and psoriasis, from cross-sectional cohorts to clinical trials, characterized by a range of different measures and parameters across the various domains of disease severity. A survey of available datasets within BIOMAP revealed the availability of a heterogeneous series of disease severity measures, both within and between studies (Appendix S1; see Supporting Information).

### Study designs and data types

Datasets available within the consortium could be broadly categorized as population-based and disease-specific, and many datasets included longitudinal follow-up. Population-based datasets were large (typically > 1000 individuals with AD/psoriasis within the population) and captured a representative range of disease severity, most individuals having less severe disease as indicated by linked health resource utilization data (e.g. healthcare/treatment use). For many of these datasets, associated biobanks provide access to genetic data. Disease-specific datasets typically provide data on multiple molecular levels (genetic, epigenetic, transcriptomic, etc.) and include multiple indicators of disease severity (disease severity scores, quality-of-life measures) recorded as continuous measures. These datasets were typically ascertained through specialist care and thus represent a more severe subset of the disease population (e.g. those requiring systemic therapy)^52^ compared with the population-based datasets.

### Classification framework for disease severity and definitions of more severe disease

The classification framework created to categorize severity measures for AD and psoriasis in relation to the agreed skin inflammation disease severity domains (symptoms, inflammatory activity and disease course) is detailed in Table 1 and Appendix S2 (see Supporting Information). Instruments/measures and definitions were prioritized using the following criteria: (i) subject to formal, international consensus as a prioritized instrument for domain of interest and (ii) expert BIOMAP consensus and/or reference standard instrument. International consensus or regulatory reference standards for more severe disease (or ‘severe’ disease) were available for symptoms and inflammatory activity, but were generally missing in relation to disease course.

**Table 1:** Atopic dermatitis and psoriasis severity classification frameworks

Prioritized instruments/measures and definitions	Skin inflammation					Impact on the individual, their family, friends and carers
	Symptoms	Inflammatory activity	Disease course			
			Disease control	Health service setting^a^	Treatment use	Quality-of-life measures
**Atopic dermatitis**	Patient-Oriented Eczema Measure (POEM) (range 0–28)^b,c,d^	Eczema area and severity index (EASI) (range 0–72)^b,c,d^	Recap of atopic eczema (RECAP) (range 0–28)^c,d,30^	Level of care received for atopic dermatitis	Type of therapy received for atopic dermatitis	Dermatology Life Quality Index (DLQI) (range 0–30)^b,c,d,17,20^
	Prioritized definition: POEM – mild: 0 ≤ *x* < 8; moderate: 8 ≤ *x* < 17; severe: 17 ≤ *x* < 28)^67–69^	Prioritized definition: EASI – clear: 0; almost clear: 0 ≤ *x* < 1; mild: 1 ≤ *x* < 7; moderate: 7 ≤ *x* < 21; severe: 21 ≤ *x* < 50; very severe: 50 ≤ *x* < 72^22,24^	Atopic Dermatitis Control Test (range 0–24)^d,31^	Prioritized discriminator: *whether affected individual is under care of specialist hospital dermatology*^c^	Prioritized discriminator: *whether affected individual is treated with systemic immunomodulating therapy*^b,17,70^	Prioritized definition: 0–1, no effect at all on patient’s life 2–5, small effect on patient’s life 6–10, moderate effect on patient’s life 11–20, very large effect on patient’s life 21–30, extremely large effect on patient’s life
**Psoriasis**	Psoriasis Symptom Scale (ordinal scale, range 0–4)^c,71^	Psoriasis Area and Severity Index (PASI) (range 0–72)^a,b^		Level of care received for psoriasis	Type of therapy received for psoriasis	DLQI (range 0–30)^a,b,17,20^
	Prioritized definition: none, 0; mild, 1; moderate, 2; severe, 3; very severe, 4	Prioritized definition: Nonsevere: PASI < 10 Severe: PASI ≥ 10^21^		Prioritized discriminator: *whether patient is under care of specialist hospital dermatology*^c^	Prioritized discriminator: *whether patient is treated with systemic immunomodulating therapy*^b,c^	Prioritized definition: 0–1, no effect at all on patient’s life 2–5, small effect on patient’s life 6–10, moderate effect on patient’s life 11–20, very large effect on patient’s life 21–30, extremely large effect on patient’s life

^a^Healthcare delivery varies across countries which may influence severity classification. Measures common to atopic dermatitis and psoriasis are highlighted in italics. Instruments/measures and definitions were prioritized using the following criteria: ^b^subject to formal, international consensus as a prioritized instrument for domain of interest;^20^ and ^c^expert BIOMarkers in Atopic Dermatitis and Psoriasis consensus and/or reference standard instrument for domain of interest. ^d^Harmonising Outcomes for Eczema core outcome set instruments.^72^

## Analytical considerations

### The relationship between disease severity measures, biomarker development and purpose

The biomarker development process comprises several steps that can be iterative, including biomarker discovery, analytical validation, clinical qualification and establishment of clinical utility.^53^ The value of different severity measures will vary depending on the stage within the biomarker development process and proposed final use of the biomarker (Table 2).^12,54^ In the biomarker discovery phase, use of a single measure or prioritized measures, typically capturing the inflammatory activity, may help to focus efforts. Subsequent evaluation across multiple comparable measures/domains provides cumulative supportive evidence for the clinical qualification and utility of candidate biomarkers and should include relevant subdomains of skin inflammation (e.g. symptoms), and both physician- and patient-reported measures. Performance of disease severity biomarkers in domains relating to impact on individuals and society may become important for clinical qualification and implementation.

**Table 2:** Purpose of disease severity biomarkers

Biomarker type	Definition	Clinical value
Diagnostic biomarker	A biomarker used to identify individuals who have severe disease at the time of measurement (e.g. serum proteomic biomarkers)	Objective measures of disease severity to facilitate comparability of baseline disease severity between study populations in observational studies and clinical trials
Monitoring biomarker	A biomarker measured repeatedly for assessing disease severity or change in severity over time (e.g. transcriptomic biomarkers associated with inflammatory activity at a point in time)	Objective measures of treatment response (clinical practice, trials)
Prognostic biomarker	A biomarker measured early in the disease course that is used to prospectively identify the likelihood of progression to disease that is more difficult to control (e.g. genetic biomarkers associated with need for systemic treatment)	Population stratification to delineate future high need populations to evaluate benefit of early intervention (disease remission, prevention of comorbidities)
Predictive biomarker	A biomarker used to prospectively identify the likely response to a treatment	Informed treatment selection; improved outcomes (more effective; fewer adverse effects)
Mechanistic biomarker	A biomarker on an established biologic pathway	May also be diagnostic, monitoring, prognostic or predictive biomarkers

### Disease severity at a point in time vs. severity over a period of time (temporality)

Identifying mechanistic pathways mediating disease severity at a point in time and over a period of time is important given the fluctuating nature of chronic inflammatory skin disease, with unpredictable flares and periods of lower activity. The molecular processes underlying each concept may be overlapping, but equally these processes may have distinct elements. Biomarkers discovered using disease severity data at a point in time (e.g. disease severity scores such as PASI or EASI) may therefore benefit from subsequent evaluation using measurements that capture disease severity over a period of time (e.g. number of disease flares over 1 year or need for systemic treatment) and vice versa. Given their relatively fixed and by definition pre-existing nature, genetic biomarkers may have better *predictive* capacity compared with dynamic epigenetic and transcriptomic biomarkers, which may be a result or consequence of disease (or its treatment).

Biomarkers that associate with disease severity measures at a point in time may serve as useful diagnostic biomarkers of disease severity (e.g. associating with current active disease) or monitoring biomarkers (which have the potential to objectively track disease activity over time). Alternatively, biomarkers that associate with disease severity over a period of time could serve as prognostic biomarkers to predict potential disease progression to less manageable disease earlier in the disease course.

### Influence of treatment on measures of skin inflammation

A high disease severity measure (e.g. PASI, EASI) on treatment may reflect ‘more severe’ underlying inflammation and/or a poor response to treatment which may, in turn, arise for several reasons. Therefore, distinguishing underlying severity from treatment effect can be very challenging. Any analysis using severity measures requires careful consideration of possible confounders and mediators (including current treatment). Some trials and registers record severity prior to treatment initiation, which may allow inference of off-treatment disease activity (e.g. how AD symptoms vary over time). Prescription and use of topical therapies is generally poorly captured, particularly in population-based datasets, and is therefore a frequently unmeasured influential factor on disease severity measures.

### Benefits and risks of dichotomizing outcomes into severe/nonsevere

Clinical decision making often requires distinct classes (normal/abnormal, treat/do not treat, etc.) favouring dichotomization in clinical research.^55^ Categorization of continuous variables can simplify both the analysis and interpretation of results in research, but this risks missing important signals; categorization may diminish statistical power and can conceal within-category information. The utility of categorizing disease severity scores should be carefully considered, accounting for the needs of the research question in any analysis. Additionally, the lack of widely accepted cutoff points for categories in both AD and psoriasis limits comparison between studies.

In psoriasis, the use of binary (severe/nonsevere) rather than traditionally used categorical severity thresholds (mild/moderate/severe) has been proposed by the IPC following a Delphi consensus exercise.^17^ Other categorization approaches have anchored proposed thresholds on Investigator’s Global Assessment categories in both AD^24^ and psoriasis.^25^

Analytical approaches and interpretation are expected to evolve within this framework and will also depend on data availability; further refinement, ethical discussions and formal international consensus will be required to agree core outcomes of disease severity for biomarker regulatory approval and to address implementation challenges.

### Availability of data

Datasets across different study types commonly capture a heterogeneous variety of disease severity measures as highlighted in the data survey. Therefore, prioritized outcome measures may frequently be unavailable for analysis. This necessitates a pragmatic approach, taking the source and nature of each measure (such as the domain of disease severity) into consideration, alongside undertaking cross-measure validation where appropriate. Initiatives such as HOME and IDEOM will be of crucial importance in driving consensus and adoption of core outcomes to increase data available for comparative studies or meta-analysis. Moreover, general efforts need to be made to ensure that there are datasets for biomarkers that are representative of global populations and that biases in the datasets are reduced.

### Conceptual and ethical considerations

Ethical and medical philosophical considerations are required with regard to describing disease severity and what constitutes ‘severe’ disease in the context of biomarker development and subsequent adoption into clinical and public health settings. Stakeholders should consider the conceptual assumptions of disease, disease severity (and related measures), in addition to how these assumptions vary across populations and settings.^56,57^ The concept of disease severity evokes a plethora of thick concepts (meaning concepts that incorporate both description and evaluation),^57,58^ including ‘wellbeing’.^59,60^

Discussions should address the various social and ethical implications that arise in biomarker research and the use of biomarkers for AD and psoriasis.^61^ Moreover, ethical debates on severity biomarkers should cover, for example, further issues of epistemic injustice^62^ and distributive justice, how severity measures should be used in healthcare priority setting^60,63–66^ and in cost-effectiveness reasoning.^63,64^ The ethical implications that certain definitions of severity might have in relation to the use of biomarkers require continual evaluation and should be an integral part of responsible biomarker research and policymaking.

## Summary, limitations and implications for future research

This review highlights the multifaceted nature of disease severity in AD and psoriasis in addition to challenges associated with studying disease and quantifying severity in the biomarker discovery pipeline. We have summarized key factors to consider (Table 3) in order to ensure that biomarker discovery efforts translate into tools with clinical utility and ethical application.

**Table 3:** Key aspects required to expedite biomarker development and validation

Consider the **stage** within the biomarker development process and **proposed use** of the biomarker when selecting disease severity measures for analysis
Evaluate candidate biomarkers **across multiple comparable severity measures/domains** to strengthen evidence of translational utility
Evaluate both **physician- and patient-**reported measures when validating biomarker associations with disease severity outcomes
Consider **influential factors** on severity measures such as treatment at the time of assessment in analyses
Explore the validity of candidate biomarkers of disease severity at a **point in time** (e.g. disease severity scores such as Psoriasis Area and Severity Index or Eczema Area and Severity Index) and disease severity over a **period of time** (e.g. number of disease flares over 1 year, or need for systemic treatment). Both have clinical utility
Consider the ethical and social implications in responsible biomarker research and implementation of disease severity biomarkers

Our framework has several limitations in relation to the following aspects: (i) methods, (ii) data sources and (iii) the system used for reaching consensus. From a methodological perspective, the literature review conducted was not systematic, and potentially overlooked some parameters or definitions of disease severity. The data sources used for mapping originated solely from European populations, thereby constraining their applicability and generalizability to diverse global demographics. The primary objective of this study was to catalyse discussions and commence the formulation of expert consensus regarding pivotal instruments and methodologies for defining severity in the realm of the biomarker development pipeline. However, to achieve broader consensus among all pertinent stakeholders, formal consensus-building efforts, inclusive of regulatory body participation, will be imperative in subsequent phases.

Our suggested framework is only a first step, and will require further discussion and modification, along with formal consensus on core disease severity outcomes where such agreement is missing. Such efforts would be expected to drive standardization on the recording of AD and psoriasis severity outcomes in future data collection and research, drive coherence and focus across BIOMAP and other consortia analyses so that mechanistic insights and future biomarkers are clinically relevant and patient-centric, and expedite biomarker qualification and approval for use.

## Supplementary Material

ljae080_Supplementary_Data

## Data Availability

All data are incorporated into the article and its online ­supplementary material.
