# Supplementary material for: Defining disease severity in atopic dermatitis and psoriasis for the application to biomarker research: an interdisciplinary perspective
Source: Br J Dermatol. 2024 Feb 29;191(1):14–23. doi: 10.1093/bjd/ljae080 (PMC11188926; doi:10.1093/bjd/ljae080)
Supplement: ljae080_Supplementary_Data [file ljae080_supplementary_data.zip › Appendix S2- Defining disease severity in atopic dermatitis and psoriasis v2.docx]

| **Atopic dermatitis (AD) severity classification framework** | | | | | | | | | | |
| --- | --- | --- | --- | --- | --- | --- | --- | --- | --- | --- |
|  | | **Skin inflammation** | | | | | | | | **Impact on the individual, their family, friends, and carers** |
|  |  | **Symptoms** | **Inflammatory activity** | | **Disease course** | | | | | **Quality of life measures** |
|  |  |  |  |  | **Disease control** | | **Health service setting ^** | | **Treatment use** |  |
| **Prioritised instruments/measures and definitions** | | Patient-Oriented Eczema Measure (POEM) [range 0–28]^a,b,⋕^  Prioritised definition:  POEM- Mild: 0≤x<8, moderate: 8≤x<17, severe: 17≤x<28) ^1, 2, 3^ | Eczema area and severity index (EASI) [range 0–72]^a,b,⋕^  Prioritised definition:  EASI- clear:0, almost clear: 0≤x<1, mild:1≤x<7, moderate:7≤x<21, severe: 21≤x<50, very severe: 50≤x<72 ^4, 5^ | | - Recap of Atopic Dermatitis (RECAP) [range 0–28] ^b⋕^ ^6^ - Atopic Dermatitis Control Test (ADCT [range 0–24]) ^7⋕^ | | Level of care received for AD  Prioritised discriminator: *whether affected individual is under care of specialist hospital dermatology* ^b^ | | Type of therapy received for AD  Prioritised discriminator: *whether affected individual is treated with systemic immuno­modulating therapy ^8, 9,^ ^b^* | *Dermatology Life Quality Index (DLQI) [range 0–30] ^a,b,⋕^ ^8, 10^*  Prioritised definition:   - 0-1 = no effect at all on patient’s life - 2-5 = small effect on patient’s life - 6-10 = moderate effect on patient’s life - 11-20 = very large effect on patient’s life - 21-30 = extremely large effect on patient’s life |
| **Other instruments/ measures** | | - Patient oriented scoring atopic dermatitis (PO-SCORAD) [range 0–83] ^11^ - Itch Numeric Rating Scale (Itch NRS) [range 0–10] ^12^ - Sleep Numerical Rating Scale (Sleep NRS) [range 0–10] | - Scoring of Atopic Dermatitis (SCORAD) [range 0–103] ^10^ - Objective Scoring of Atopic Dermatitis (OSCORAD) ^13^ - *Investigator’s global assessment (IGA) [ordinal scale, multiple scale ranges used] ^14^* - Validated Investigator Global Assessment scale for Atopic Dermatitis (vIGA-AD) [ordinal scale, range- 0: clear, 1: almost clear, 2: mild, 3: moderate, 4: severe] ^15^ - *Patient global assessment [ordinal scale, range- 0: clear, 1: almost clear, 2: mild, 3: moderate, 4: severe] ^16^* - *Body surface area (BSA) [range 0-100%] [severe:* ≥10*%, non-severe: <10%]* - *Product of IGA and body surface area (IGAxBSA) [range 0–400] ^17^* - TIS score (Three Item Severity score) [range 0-3] ^18^ | | - Atopic Dermatitis Control Test (ADCT [range 0–24]) ^7⋕^ - Number of weeks atopic dermatitis has been well controlled in the last 12 weeks | | - *Tertiary level subspecialist care* - *Emergency department attendance* - *Inpatient hospital care* - *Intensive care* | | - Use of potent/ super-potent topical steroids - *Use of phototherapy* - *Treatment Satisfaction Questionnaire for Medication (TSQM)^19^* - *Questionnaire on Cumulative Life Course Impairment in skin diseases (DermCLCI-r and DermCLCI-p)^20^* | - *Children’s dermatology Life Quality Index (CDLQI) [range 0–30]^⋕^* - *Teenager’s Quality of Life (T-QoL) [range 0–36] ^21^* - *Family Dermatology Life Quality Index (FDLQI) [range 0–30] ^22^* - *EQ5D [range 0–1] ^23^* - *Major Life Changing Decision Profile (MLCDP) [range 0–32] ^24^* - *Family Reported Outcome Measure (FROM-16) [range 0–32] ^25^*   AD-specific measures:   - Infant’s Dermatitis Quality of Life (IDQOL) [range 0-30] ^⋕^ ^26^ - Dermatitis Family Impact Questionnaire (DFI) [range 0-30] ^27^ |
| **Quantif-ication metric** | **Measures at a point in time** | Continuous (including integer representation of ordinal scales)  OR  Categorical (ordinal) representation of continuous measure (e.g., POEM- mild: 0≤x<8, moderate: 8≤x<17, severe: 17≤x<28) ^1, 2, 3^  OR  Dichotomisation of continuous measure  OR  Absolute/relative change with respect to baseline | Continuous (including integer representation of ordinal scales)  OR  Categorical (ordinal) representation of continuous measure:  E.g.,   - EASI- clear: 0, almost clear: 0≤x<1, mild: 1≤x<7, moderate: 7≤x<21, severe: 21≤x<50, very severe: 50≤x<72.0 ^4, 5^ - SCORAD- mild: 0≤x<29.0, moderate: 29.1≤x<49.0, severe: 49.1≤x<103 ^1, 11, 28^ - OSCORAD- mild: 0≤x<24.0, moderate: 24.1≤x<38, severe: 38.1≤x<83 ^28, 29^)   OR  Binary dichotomisation of continuous measure (e.g., non-severe: EASI<23, severe: EASI≥23)  OR  Absolute/relative change with respect to baseline | Continuous (including integer representation of ordinal scales)  OR  Categorical (ordinal) representation of continuous measure  OR  Dichotomisation of continuous measure  OR  Absolute/relative change with respect to baseline | | Binary (Non-severe- AD managed outside of higher-level care, Severe- currently receiving higher level care for AD) | | Binary (Non-severe- not currently receiving higher level treatment for AD, Severe- currently receiving higher level treatment for AD) | | Continuous (including integer representation of ordinal scales)  OR  Categorical (ordinal) representation of continuous measure  OR  Dichotomisation of continuous measure (e.g., non-severe: DLQI<10, severe: DLQI≥10)^30^  OR  Absolute/relative change with respect to baseline |
|  | **Measures over a period of time (e.g., >12 months)** | Average measures  OR  Maximum measures  OR  Area under the curve measures for >2 observations | Average measures  OR  Maximum measures  OR  Area under the curve measures for >2 observations | Average measures  OR  Maximum measures  OR  Area under the curve measures for >2 observations | | Continuous (e.g., number of attendances to above healthcare settings over time)  OR  Binary (non-severe/active- no utilization of higher level of care, severe- ≥1 instance of requiring higher level of care) (62) *‡* | | Continuous (e.g., duration of systemic treatment)  OR  Binary (non-severe/active- no, severe- yes) | | Average measures  OR  Maximum measures  OR  Area under the curve measures for >2 observations |

*^Healthcare delivery varies across countries which may influence severity classification*

**Measures common to atopic dermatitis and psoriasis are highlighted in italics. Instruments/measures and definitions were prioritised using the following criteria: a. Subject to formal, international consensus as a prioritised instrument for domain of interest^10^ b.* *Expert BIOMAP consensus and/ or reference standard instrument for domain of interest*

*^⋕^ HOME Core Outcome Set instruments^31^*

*‡ Patient classified as ‘severe’ AD- once patient utilises higher level care, irrespective of subsequent pattern of healthcare utilisation*

| **Psoriasis severity classification framework** | | | | | | | | |
| --- | --- | --- | --- | --- | --- | --- | --- | --- |
|  | | **Skin inflammation** | | | | **Impact on the individual, their family, friends, and carers** | | |
|  |  | **Symptoms** | **Inflammatory activity** | **Disease course** | | | **Quality of life measures** |  |
|  |  |  |  | **Health service setting ^** | **Treatment use** | |  |  |
| **Prioritised instruments/measures and definitions** | | Psoriasis symptom scale (PSS) [ordinal scale, range- 0–4]^32, b^  Prioritised definition:  None:0, Mild:1, Moderate:2, Severe:3, Very severe:4 | Psoriasis area and severity index (PASI) [range 0–72] ^a,b^  Prioritised definition:   - non-severe: PASI<10 - severe: PASI≥10^30^ | Level of care received for psoriasis  Prioritised discriminator: *whether patient is under care of specialist hospital dermatology* ^b^ | Type of therapy received for psoriasis  Prioritised discriminator: *whether patient is treated with systemic immuno­modulating therapy ^a,b^* | | *Dermatology Life Quality Index (DLQI) [range 0–30]^8, 10^ ^a,b^*  Prioritised definition:   - 0-1 = no effect at all on patient’s life - 2-5 = small effect on patient’s life - 6-10 = moderate effect on patient’s life - 11-20 = very large effect on patient’s life - 21-30 = extremely large effect on patient’s life |  |
| **Other instruments/. measures** | |  | - *Body surface area (BSA) [range 0-100%] [severe:* ≥10*%, non-severe: <10%]* - Psoriasis global assessment (PGA) [ordinal scale, multiple scale ranges used] ^33^ - *Patient global assessment (ordinal scale, 5,6,7 point scale ranges used) ^34^* - *Investigator’s global assessment (IGA) [ordinal scale, range- 0: clear, 1: almost clear, 2: mild, 3: moderate, 4: severe, 5: very severe] ^35^* - Simplified Psoriasis Area Severity Index (SPASI) [range 0–36] ^36, 37^ *[severe:* ≥12*, non-severe: <12]* - Simplified Psoriasis Index (SPI) [range 0–50 ^38^ - Patient-oriented Psoriasis area and severity index (PO-PASI) [range 0–72] ^39^ - Scalp specific Investigator’s Global Assessment (ss IGA) ) [range 0–5] ^40^ - Hand and/or foot PGA (hf-PGA) [range 0–5] ^40^ - Nail Psoriasis Severity Index (NAPSI) [range 0–32] ^41^ - Palmoplantar Pustulosis Area and Severity Index (PPPASI) [range 0–72] ^42^ - Palmoplantar Pustular Psoriasis Pustule count - Generalized Pustular Psoriasis Area and Severity Index (GPPASI) [range 0–72] ^43^ | - *Tertiary level subspecialist care* - *Emergency department attendance* - *Inpatient hospital care* - *Intensive care* | - *Use of phototherapy (UVB/PUVA)* - *Treatment Satisfaction Questionnaire for Medication (TSQM)^19^* - *Questionnaire on Cumulative Life Course Impairment in skin diseases (DermCLCI-r and DermCLCI-p)^20^* | | General measures:   - *Children’s dermatology Life Quality Index (CDLQI) [range 0–30]* - *Teenager’s Quality of Life (T-QoL) [range 0–36] ^21^* - *EQ5D [range 0–1] ^23^* - *Family Dermatology Life Quality Index (FDLQI) [range 0–30] ^22^* - *Major Life Changing Decision Profile (MLCDP) [range 0–32] ^24^* - *Family Reported Outcome Measure (FROM-16) [range 0–32] ^25^*   Psoriasis-specific measures:   - Psoriasis disability index [range 0–45] ^44^ - Psoriasis Family Index (PFI-14) [range 0–42] ^45^ |  |
| **Quantif-ication metric** | **Measures at a point in time** | Binary dichotomization of ordinal measure  OR  Categorical (ordinal) representation of continuous measure  OR  Absolute/relative change with respect to baseline | Continuous (including integer representation of ordinal scales)  OR  Dichotomization of ordinal measure:  E.g.,   - non-severe: PASI<10 - severe: PASI≥10) ^30^   OR  Categorical (ordinal) representation of continuous measure  E.g.,   - PASI- mild: 0≤x<7, moderate: 7≤x<12, severe: x>12 ^46^, - BSA- mild: BSA 0≤x<3% moderate: BSA 3%≤x<10% severe: x≥10%   OR  Absolute/relative change with respect to baseline | Binary (non-severe- psoriasis managed outside of higher level care, severe- patient receiving higher level care for psoriasis) | Binary (non-severe- not currently receiving higher level treatment for psoriasis, severe- currently receiving higher level treatment for psoriasis) | | Continuous (including integer representation of ordinal scales)  OR  Categorical (ordinal) representation of continuous measure  OR  Dichotomisation of continuous measure (e.g., non-severe- DLQI<10, severe- DLQI≥10) ^30^  OR  Absolute/relative change with respect to baseline |  |
|  | **Measures over a period of time (e.g., >12 months)** | Average measures  OR  Maximum measures  OR  Area under the curve measures | Average measures  OR  Maximum measures  OR  Area under the curve measures | Continuous (e.g., number of attendances to above healthcare settings over time)  OR  Binary (non-severe- no utilization of higher level of care, severe- >1 instance of requiring higher level of care) ‡ | Continuous (e.g., duration of systemic treatment)  OR  Binary (non-severe- no, severe- yes) | | Average measures  OR  Maximum measures  OR  Area under the curve measures |  |

*^Healthcare delivery varies across countries which may influence severity classification*

**Measures common to atopic dermatitis and psoriasis are highlighted in italics. Instruments/measures and definitions were prioritised using the following criteria:- a. Subject to formal, international consensus as prioritised instrument for domain of interest b.* *Expert BIOMAP consensus and/ or reference standard instrument for domain of interest*

*‡ Patient classified as ‘severe’ psoriasis- once patient utilises higher level care, irrespective of subsequent pattern of healthcare utilisation*

**References**

1. Yang YB, Lynde CW, Fleming P. Common Atopic Dermatitis Rating Scales: A Practical Approach and Brief Review [Formula: see text]. J Cutan Med Surg. 2020;24(4):399-404.

2. Charman CR, Venn AJ, Ravenscroft JC, Williams HC. Translating Patient-Oriented Eczema Measure (POEM) scores into clinical practice by suggesting severity strata derived using anchor-based methods. Br J Dermatol. 2013;169(6):1326-32.

3. Charman CR, Venn AJ, Williams HC. The patient-oriented eczema measure: development and initial validation of a new tool for measuring atopic eczema severity from the patients' perspective. Arch Dermatol. 2004;140(12):1513-9.

4. Hanifin JM, Baghoomian W, Grinich E, Leshem YA, Jacobson M, Simpson EL. The Eczema Area and Severity Index-A Practical Guide. Dermatitis. 2022;33(3):187-92.

5. Leshem YA, Hajar T, Hanifin JM, Simpson EL. What the Eczema Area and Severity Index score tells us about the severity of atopic dermatitis: an interpretability study. British Journal of Dermatology. 2015;172(5):1353-7.

6. Howells LM, Chalmers JR, Gran S, Ahmed A, Apfelbacher C, Burton T, et al. Development and initial testing of a new instrument to measure the experience of eczema control in adults and children: Recap of atopic eczema (RECAP). Br J Dermatol. 2020;183(3):524-36.

7. Simpson E, Eckert L, Gadkari A, Mallya UG, Yang M, Nelson L, et al. Validation of the Atopic Dermatitis Control Tool (ADCT©) using a longitudinal survey of biologic-treated patients with atopic dermatitis. BMC Dermatol. 2019;19(1):15.

8. Strober B, Ryan C, van de Kerkhof P, van der Walt J, Kimball AB, Barker J, et al. Recategorization of psoriasis severity: Delphi consensus from the International Psoriasis Council. J Am Acad Dermatol. 2020;82(1):117-22.

9. Silverwood RJ, Mansfield KE, Mulick A, Wong AYS, Schmidt SAJ, Roberts A, et al. Atopic eczema in adulthood and mortality: UK population-based cohort study, 1998-2016. J Allergy Clin Immunol. 2021;147(5):1753-63.

10. <http://www.homeforeczema.org/>. HOME for eczema: Harmonising Outcome Measures for Eczema [Available from: <http://www.homeforeczema.org/>.

11. Stalder JF, Barbarot S, Wollenberg A, Holm EA, De Raeve L, Seidenari S, et al. Patient-Oriented SCORAD (PO-SCORAD): a new self-assessment scale in atopic dermatitis validated in Europe. Allergy. 2011;66(8):1114-21.

12. Silverberg JI, DeLozier A, Sun L, Thyssen JP, Kim B, Yosipovitch G, et al. Psychometric properties of the itch numeric rating scale, skin pain numeric rating scale, and atopic dermatitis sleep scale in adult patients with moderate-to-severe atopic dermatitis. Health Qual Life Outcomes. 2021;19(1):247.

13. Oranje AP, Glazenburg EJ, Wolkerstorfer A, de Waard-van der Spek FB. Practical issues on interpretation of scoring atopic dermatitis: the SCORAD index, objective SCORAD and the three-item severity score. Br J Dermatol. 2007;157(4):645-8.

14. Futamura M, Leshem YA, Thomas KS, Nankervis H, Williams HC, Simpson EL. A systematic review of Investigator Global Assessment (IGA) in atopic dermatitis (AD) trials: Many options, no standards. J Am Acad Dermatol. 2016;74(2):288-94.

15. Simpson E, Bissonnette R, Eichenfield LF, Guttman-Yassky E, King B, Silverberg JI, et al. The Validated Investigator Global Assessment for Atopic Dermatitis (vIGA-AD): The development and reliability testing of a novel clinical outcome measurement instrument for the severity of atopic dermatitis. J Am Acad Dermatol. 2020;83(3):839-46.

16. Grinich EE, Pawlitschek T, Simpson EL. Reporting of Patient Global Assessments in Atopic Dermatitis Randomized Controlled Trials. Dermatitis. 2020;31(1):e7-e9.

17. Suh TP, Ramachandran D, Patel V, Jackson KL, Rangel SM, Fishbein AB, et al. Product of Investigator Global Assessment and Body Surface Area (IGAxBSA): A practice-friendly alternative to the Eczema Area and Severity Index to assess atopic dermatitis severity in children. J Am Acad Dermatol. 2020;82(5):1187-94.

18. Wolkerstorfer A, de Waard van der Spek FB, Glazenburg EJ, Mulder PG, Oranje AP. Scoring the severity of atopic dermatitis: three item severity score as a rough system for daily practice and as a pre-screening tool for studies. Acta Derm Venereol. 1999;79(5):356-9.

19. Atkinson MJ, Sinha A, Hass SL, Colman SS, Kumar RN, Brod M, et al. Validation of a general measure of treatment satisfaction, the Treatment Satisfaction Questionnaire for Medication (TSQM), using a national panel study of chronic disease. Health Qual Life Outcomes. 2004;2:12.

20. Braren-von Stülpnagel CC, Augustin M, Westphal L, Sommer R. Development of Measurement Tools to Assess Cumulative Life Course Impairment in Patients with Chronic Skin Diseases. J Eur Acad Dermatol Venereol. 2023.

21. Basra MKA, Salek MS, Fenech D, Finlay AY. Conceptualization, development and validation of T-QoL(©) (Teenagers' Quality of Life): a patient-focused measure to assess quality of life of adolescents with skin diseases. Br J Dermatol. 2018;178(1):161-75.

22. Basra MK, Sue-Ho R, Finlay AY. The Family Dermatology Life Quality Index: measuring the secondary impact of skin disease. Br J Dermatol. 2007;156(3):528-38.

23. Rabin R, de Charro F. EQ-5D: a measure of health status from the EuroQol Group. Ann Med. 2001;33(5):337-43.

24. Bhatti ZU, Salek SS, Bolton CE, George L, Halcox JP, Jones SM, et al. The development and validation of the major life changing decision profile (MLCDP). Health Qual Life Outcomes. 2013;11:78.

25. Golics CJ, Basra MK, Finlay AY, Salek S. The development and validation of the Family Reported Outcome Measure (FROM-16)© to assess the impact of disease on the partner or family member. Qual Life Res. 2014;23(1):317-26.

26. Lewis-Jones MS, Finlay AY, Dykes PJ. The Infants' Dermatitis Quality of Life Index. Br J Dermatol. 2001;144(1):104-10.

27. Lawson V, Lewis-Jones MS, Finlay AY, Reid P, Owens RG. The family impact of childhood atopic dermatitis: the Dermatitis Family Impact Questionnaire. Br J Dermatol. 1998;138(1):107-13.

28. Chopra R, Vakharia PP, Sacotte R, Patel N, Immaneni S, White T, et al. Relationship between EASI and SCORAD severity assessments for atopic dermatitis. J Allergy Clin Immunol. 2017;140(6):1708-10.e1.

29. Severity scoring of atopic dermatitis: the SCORAD index. Consensus Report of the European Task Force on Atopic Dermatitis. Dermatology. 1993;186(1):23-31.

30. Finlay AY. Current severe psoriasis and the rule of tens. Br J Dermatol. 2005;152(5):861-7.

31. Williams HC, Schmitt J, Thomas KS, Spuls PI, Simpson EL, Apfelbacher CJ, et al. The HOME Core outcome set for clinical trials of atopic dermatitis. J Allergy Clin Immunol. 2022;149(6):1899-911.

32. Rentz AM, Skalicky AM, Burslem K, Becker K, Kaschinski D, Esser D, et al. The content validity of the PSS in patients with plaque psoriasis. J Patient Rep Outcomes. 2017;1(1):4.

33. Mahil SK, Wilson N, Dand N, Reynolds NJ, Griffiths CEM, Emsley R, et al. Psoriasis treat to target: defining outcomes in psoriasis using data from a real-world, population-based cohort study (the British Association of Dermatologists Biologics and Immunomodulators Register, BADBIR). Br J Dermatol. 2020;182(5):1158-66.

34. Perez-Chada LM, Salame NF, Ford AR, Duffin KC, Garg A, Gottlieb AB, et al. Investigator and Patient Global Assessment Measures for Psoriasis Clinical Trials: A Systematic Review on Measurement Properties from the International Dermatology Outcome Measures (IDEOM) Initiative. Am J Clin Dermatol. 2020;21(3):323-38.

35. Langley RG, Feldman SR, Nyirady J, van de Kerkhof P, Papavassilis C. The 5-point Investigator's Global Assessment (IGA) Scale: A modified tool for evaluating plaque psoriasis severity in clinical trials. J Dermatolog Treat. 2015;26(1):23-31.

36. Louden BA, Pearce DJ, Lang W, Feldman SR. A Simplified Psoriasis Area Severity Index (SPASI) for rating psoriasis severity in clinic patients. Dermatol Online J. 2004;10(2):7.

37. Cao D, Shen M, Chen X, Xiao Y, Lu W, Luo Y, et al. Validation of a simple measure of psoriasis severity based on a longitudinal study of Chinese patients. Eur J Dermatol. 2020;30(6):674-9.

38. Chularojanamontri L, Griffiths CE, Chalmers RJ. The Simplified Psoriasis Index (SPI): a practical tool for assessing psoriasis. J Invest Dermatol. 2013;133(8):1956-62.

39. Montes de Oca Pedrosa A, Oakley A, Rogers J, Rangaitaha Epiha M. PASI vs PO-PASI: Patient-Oriented PASI (PO-PASI) is comparable to clinician score PASI. Australas J Dermatol. 2022;63(1):e67-e70.

40. Foley P, Gordon K, Griffiths CEM, Wasfi Y, Randazzo B, Song M, et al. Efficacy of Guselkumab Compared With Adalimumab and Placebo for Psoriasis in Specific Body Regions: A Secondary Analysis of 2 Randomized Clinical Trials. JAMA Dermatol. 2018;154(6):676-83.

41. Rich P, Scher RK. Nail Psoriasis Severity Index: a useful tool for evaluation of nail psoriasis. J Am Acad Dermatol. 2003;49(2):206-12.

42. Bhushan M, Burden AD, McElhone K, James R, Vanhoutte FP, Griffiths CE. Oral liarozole in the treatment of palmoplantar pustular psoriasis: a randomized, double-blind, placebo-controlled study. Br J Dermatol. 2001;145(4):546-53.

43. Navarini AA, Burden AD, Capon F, Mrowietz U, Puig L, Köks S, et al. European consensus statement on phenotypes of pustular psoriasis. J Eur Acad Dermatol Venereol. 2017;31(11):1792-9.

44. FINLAY AY, KHAN GK, LUSCOMBE DK, SALEK MS. Validation of Sickness Impact Profile and Psoriasis Disability Index in psoriasis. British Journal of Dermatology. 1990;123(6):751-6.

45. Eghlileb AM, Basra MK, Finlay AY. The psoriasis family index: preliminary results of validation of a quality of life instrument for family members of patients with psoriasis. Dermatology. 2009;219(1):63-70.

46. Schmitt J, Wozel G. The psoriasis area and severity index is the adequate criterion to define severity in chronic plaque-type psoriasis. Dermatology. 2005;210(3):194-9.
